# Metabolic Dysfunction-Associated Steatotic Liver Disease and Sarcopenia: Influence of Habitual Food

**DOI:** 10.3390/diagnostics15212711

**Published:** 2025-10-27

**Authors:** Naiade S. Almeida, Raquel Rocha, Carla Daltro, Helma P. Cotrim

**Affiliations:** 1 Post-Graduated Program in Medicine and Heath (PPGMS), Bahia Medicine School, Universidade Federal da Bahia, Salvador 40110-060, Bahia, Brazil; naiade.silveira@hotmail.com; 2 Department of Nutrition Sciences, Nutrition School, Universidade Federal da Bahia, Salvador 40110-060, Bahia, Brazil; raquelrocha2@yahoo.com.br (R.R.); carlahcdaltro@gmail.com (C.D.)

**Keywords:** metabolic dysfunction-associated steatotic liver disease, MASLD, food consumption, sarcopenia, diets

## Abstract

**Background**: Sarcopenia is a clinical condition linked to various liver diseases, including metabolic dysfunction-associated steatotic liver disease (MASLD). MASLD includes a spectrum from steatosis to steatohepatitis, which may progress to fibrosis, cirrhosis, and hepatocellular carcinoma. The influence of dietary habits and nutrient intake on MASLD and its progression is well-established. However, the association between dietary consumption and sarcopenia in MASLD patients remains underexplored. This study evaluated whether there is an association between sarcopenia and habitual food consumption in MASLD patients. **Methods:** A cross-sectional study was conducted with outpatients diagnosed with MASLD. Sarcopenia was defined based on the 2019 EWGSOP2 criteria. Dietary intake was assessed using three 24 h recalls per patient, with intrapersonal variance corrected using the Multiple Source Method (MSM) software (Version 1.0.1). Steatosis was diagnosed via upper abdominal ultrasound, and the Fibrosis-4 Index (FIB-4) was used to assess hepatic fibrosis. **Results:** MASLD patients (*n* = 76) were evaluated. The mean age was 52.9 (SD, 12.0) years, and 75.0% were female. Two had sarcopenia, and 27.6% (*n* = 21) had probable sarcopenia (characterized by low muscle strength only). Among probable sarcopenia, F1-F2 were observed in 61.9%, and 23.8% had indeterminate FIB-4 grades. Calcium intake was lower among patients with probable sarcopenia than those no sarcopenia (*p* = 0.04). **Conclusions:** In these MASLD patients, only two patients were diagnosed with sarcopenia, and around a third had probable sarcopenia. The majority of MASLD patients with lower calcium, energy, and protein intake, but only lower calcium intake in those with probable sarcopenia.

## 1. Introduction

The metabolic dysfunction-associated steatotic liver disease (MASLD) [1], formerly known as non-alcoholic fatty liver disease (NAFLD), has an estimated global prevalence of 30%, making it one of the most prevalent chronic liver diseases [2]. The onset of MASLD is strongly associated with the presence of metabolic syndrome components, such as central obesity, insulin resistance (IR), type II diabetes mellitus, and dyslipidemia [3].

Sarcopenia, defined by the European Working Group on Sarcopenia in Older People (EWGSOP2), is characterized by the progressive loss of muscle strength, muscle mass quantity/quality, and physical performance [4] and is recognized as a progressive disease associated with liver disease [5].

MASLD and sarcopenia share abnormal pathophysiological mechanisms, including IR, systemic inflammation, nutritional deficiencies, and physical inactivity, suggesting a bidirectional relationship between these two clinical conditions. However, the specific mechanism of this association remains unclear [6,7,8,9].

As a multifactorial disease, MASLD is influenced by various factors, including dietary habits. Currently, the available guidelines for managing MASLD agree on the treatment of diet and physical exercise [10,11,12].

The dietary intake in individuals with MASLD according to the diagnosis of sarcopenia is still not well understood. Some studies that have evaluated dietary habits in this population use various methodologies for diagnosing sarcopenia, which results in differing prevalences and, consequently, differences in the association between dietary intake and sarcopenia [13,14].

The present study evaluated whether there is an association between sarcopenia and habitual food consumption in individuals with MASLD.

## 2. Patients and Methods

### 2.1. Study Design and Population

A cross-sectional study was conducted at the Steatohepatitis Outpatient Clinic of the University Hospital Professor Edgard Santos, Federal University of Bahia, Brazil. Individuals of both sexes, aged over 18 years, with hepatic steatosis confirmed by abdominal ultrasound and a history of occasional or no ethanol consumption (<140 g of ethanol per week), were included from June 2021 to April 2023.

### 2.2. Clinical Evaluation

All cases had MASLD diagnosis. Individuals diagnosed with other liver diseases (hepatitis A, B, C, autoimmune disease, Wilson’s disease, and hemochromatosis), hypothyroidism, pregnant and lactating women, those with abdominal tumors, recent abdominal surgeries, or any physical limitations that would impair anthropometric evaluation were not included.

### 2.3. Abdominal Ultrasound

All patients underwent upper abdomen ultrasound by a single evaluator to measure intrahepatic fat [15] in a specialized clinic, using the Xario 100 Canon Medical Systems device^®^ (Canon Medical Systems do Brasil Ltda, Barueri, Brazil).

### 2.4. Fibrosis Assessment

The Fibrosis-4 Index (FIB-4) was used to assess the presence or absence of advanced fibrosis. The Fibrosis-4 Index (FIB-4) considered the absence of advanced fibrosis as FIB-4 < 1.45; indeterminate fibrosis as FIB-4 between 1.45 and 3.24; and advanced fibrosis as FIB-4 ≥ 3.25 [16,17].

### 2.5. Clinical, Demographic, and Dietary Assessment

Demographic data were collected using a questionnaire developed and standardized by the researchers to characterize the sample.

The assessment of food consumption was conducted using three 24 h dietary recalls (R24h) for each patient, with the first being in person at the time of the interview and the second and/or third by phone, including one weekend day [18,19]. All phone calls were made by nutrition interns who had received prior training. During data collection, in-person and by phone, the five steps recommended by the multiple-pass method [20] were used. Photographic albums with images of household measurements and food portions were also used during the in-person interview. Among the patients included in the study, some had already received nutritional guidance.

The household measurements of each food item or preparation were converted into grams (g) or milliliters (mL). Subsequently, the preparations were broken down based on standardized recipes, and the ingredients were converted into grams or milliliters [21,22]. After this conversion, the DietBox software (online version) was utilized to calculate the nutritional composition of the foods. Calculation prioritized the reference tables from the Brazilian Food Composition Table (TACO) [23], followed by the Brazilian Institute of Geography and Statistics (IBGE) [24], and lastly, Philippi [25]. This process determined the energy values and macronutrient and micronutrient content for all items recorded in the dietary recalls, enabling the analysis of both global and individual consumption.

The dietary intake information included energy, carbohydrates, proteins, total lipids, polyunsaturated, monounsaturated and saturated fats, dietary fiber, calcium, iron, phosphorus, zinc, selenium, and magnesium. Energy intake was categorized as adequate when the value was between 20 and 25 kcal/kg/body weight/day [26]. Protein consumption was evaluated in grams/kilogram of body weight per day, being considered low (<1.2 g/kg body weight) or adequate (≥1.2 g/kg body weight) [12].

### 2.6. Anthropometric and Clinical Assessment

Anthropometric measurements were taken in duplicate by a trained and standardized team. Body weight (kg) and height (cm) were measured with participants wearing light clothing and no shoes, using a digital scale with a 100 g resolution and a stadiometer with 0.5 cm. Waist circumference (WC) was measured at the midpoint between the iliac crest and the lower rib margin [27,28].

Body mass index (BMI) was calculated using the formula weight (kg)/height^2^ (m^2^) [28]. For adult patients, the classification was eutrophic (BMI 18.5 kg/m^2^ to 24.9 kg/m^2^), overweight (BMI 25.0 kg/m^2^ to 29.9 kg/m^2^) and obesity (BMI ≥ 30.0 kg/m^2^), and for elderly patients, adequate weight (BMI ≥ 23.0 kg/m^2^ to <28.0 kg/m^2^), overweight (BMI ≥ 28.0 kg/m^2^ to <30.0 kg/m^2^) and obesity (BMI ≥ 30.0 kg/m^2^) [28,29].

For the classification of WC, adult individuals were classified as having central obesity if WC values were ≥94 cm for men and ≥80 cm for women [30]. For elderly people, WC values > 102 cm for men and >88 cm for women indicate central obesity.

Metabolic syndrome was classified according to the International Diabetes Federation [31].

### 2.7. Sarcopenia Assessment

All patients underwent a questionnaire, anthropometric, and sarcopenia assessment in the first interview after consenting to participate in the research.

Based on the 2019 European Consensus (EWGSOP2) [4], the diagnosis of probable sarcopenia was considered when there was low muscle strength associated with normal muscle mass; sarcopenia was confirmed in the presence of both low muscle strength and low muscle mass; severe sarcopenia was diagnosed when low physical performance was present alongside the other parameters (muscle strength and mass) (Table 1).

#### 2.7.1. Muscle Mass

Muscle mass was assessed by calculating appendicular skeletal muscle mass (ASMM) using the prediction equation proposed by Sergi and colleagues [32], which takes into account data on weight in kg, height in cm, gender, resistance values, and reactance in ohms.

The resistance value was obtained through bioelectrical impedance analysis (BIA) using a tetrapolar Biodynamics^®^ device (Biodynamics Corporation, Seatle, WA, USA), model 450. The technique and prior procedures were conducted according to Kyle and colleagues [33]. From the ASMM, the appendicular skeletal muscle mass index (IASMM) was calculated using the equation ASMM/height^2^ [4].

Low muscle mass was defined according to the cutoff points of <5.5 kg/m^2^ for women and <7 kg/m^2^ for men [34].

#### 2.7.2. Muscle Strength

Muscle strength was assessed using the maximum handgrip strength test with the portable SAEHAN Spring Hand Dynamometer (Smedley-Type SH5002, SAEHAN Corporation, Gyeongnam, Republic of Korea), which has a measurement range of 0–100 kg-force (kg/f).

The measurement was repeated twice with the dominant hand, with a one-minute rest between each measurement. The average of the measurements was then considered for analysis. The cutoff point adopted was <27 kg/f for men and <16 kg/f for women [4].

#### 2.7.3. Physical Performance

Usual gait speed was measured in meters per second (m/s). The patient walked a distance of four meters on a flat, level surface at their usual walking speed. The test was repeated twice, and the shorter time was used for analysis. Individuals with a gait speed ≤ 0.8 m/s were assessed as having reduced gait speed or poor physical performance [4].

### 2.8. Assessment of Physical Activity Level

The short version of the International Physical Activity Questionnaire (IPAQ) was used to assess the level of physical activity [35]. Individuals were divided into five categories proposed by the IPAQ: 1. Sedentary: did not perform any physical activity for at least 10 continuous minutes per week; 2. Inadequately active B: did not meet any of the recommendation criteria regarding frequency or duration. Ex: frequency of <5x per week or <150 min/week; 3. Inadequately active A: performed physical activity at least 5 days/week or a total duration of 150 min/week; 4. Active: met the recommendations of vigorous activity ≥3 days/week and ≥20 min per session; or moderate activity or walking: ≥5 days/week and ≥30 min per session; or any activity combined: ≥5 days/week and ≥150 min/week (walking + moderate + vigorous); 5. Very active: met the recommendations of vigorous activity: ≥5 days/week and ≥30 min per session; or vigorous activity: ≥3 days/week and ≥20 min per session + moderate activity and/or walking: ≥5 days/week and ≥30 min per session.

The individuals were classified into two groups: sedentary, including those who were sedentary and insufficiently active (B); and physically active, which included those classified as insufficiently active (A), active, and very active, according to the IPAQ classification.

### 2.9. Statistical Analysis

The statistical software Statistical Package for the Social Science^®^ (SPSS) version 20.0 was used for the tabulation and data analysis. The results of sample characterization and macronutrient and micronutrient intake were presented as follows: categorical variables were expressed as absolute and relative frequencies, while continuous variables were expressed as mean and standard deviation, median, and interquartile range (IQR), depending on the nature of the variable. The normality of the variables was assessed by examining the histogram, the quantile–quantile plot, the Shapiro–Wilk test, and the difference between the mean and median values.

The data on dietary intake had the intrapersonal variance of each food and nutrient corrected using statistical modeling techniques incorporated in the Multiple Source Method (MSM) software (version 1.0.1, 2011) [36]. Subsequently, the residual method was used to adjust nutrient consumption by energy [37].

Pearson’s chi-square and Fisher’s exact tests were used to compare qualitative variables. The power of the association was assessed by Cramér’s V coefficient. The Mann–Whitney U test was used for comparisons of non-normal continuous variables between groups with no sarcopenia and with probable sarcopenia. Effect sizes were calculated using Rosenthal’s r (r = Z/√N) and interpreted according to Cohen’s (1988) [38] criteria: 0.10 (small), 0.30 (medium), 0.50 (large). Additionally, the probability of superiority (PS) was calculated to estimate the chance of an individual in one group presenting higher values than the other group. A *p*-value of <0.05 was considered statistically significant.

## 3. Results

### 3.1. Characteristics of the Studied Population

**Figure 1:**
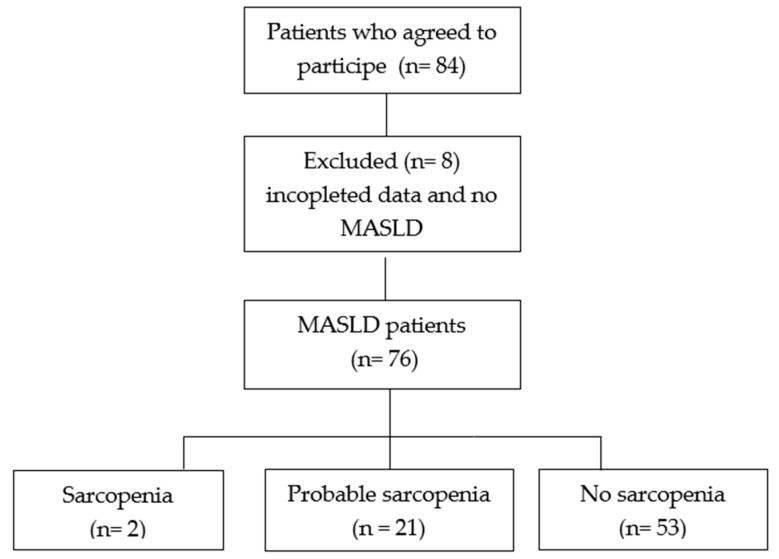
Flowchart of the cross-sectional study. MASLD, metabolic dysfunction-associated steatotic liver disease.

The sample consisted of 76 patients with MASLD. Only two patients were diagnosed with sarcopenia, and 21 (27.6%) with probable sarcopenia (Figure 1).

The mean age was (SD) 52.9 (12.0) years. The median income was USD 242.4 (This value represents 91.81% of the Brazilian minimum wage in 2023) (220.00–440.00). The majority of the patients (75.0%) were female, (63.2%) were obese, (87.8%) had an elevated waist circumference, (64.0%) had metabolic syndrome, and (65.3%) had steatosis grades II and III. When assessed by FIB-4, the degree of hepatic fibrosis was observed in 5.3% (F3/F4).

Patients with sarcopenia were excluded from the statistical analyses to facilitate better interpretation of the results. There was no statistically significant difference in sociodemographic, clinical, and anthropometric variables between individuals without sarcopenia and those with probable sarcopenia. The results did not show a statistically significant association between the variables, with virtually no effect (Cramér’s V < 0.1), suggesting that, if any association exists, it is clinically irrelevant for nutritional monitoring, metabolic syndrome, BMI, and waist circumference. Although not statistically significant, the observed effect size (Cramér’s V, 0.3–0.5) suggests a clinically relevant association with the sex variable alone. For the sex variable, the lack of significance may be due to insufficient sample size, rather than the absence of an effect (Table 2).

### 3.2. Characteristic of Dietary Intake vs. Probable Sarcopenia Diagnosis

Only calcium intake was lower among patients with probable sarcopenia (*p* = 0.04). Low energy and protein intake were observed in both groups, with no differences in the intake of macronutrients and other micronutrients (Table 3). The analysis revealed directional consistency unanimously favorable to the group with probable sarcopenia, but with only one analysis reaching individual statistical significance (*p* < 0.05). The predominance of negligible and small effects, coupled with a mean probability of superiority of 58%, suggests a modest but inconsistent advantage in terms of statistical significance. These results indicate a promising directional pattern that warrants further investigation with an increased sample size, but in the current context, present limited evidence.

### 3.3. Association Between Clinical, Anthropometric, and Dietary Intake Variables According to Probable Sarcopenia Diagnosis

In the adjusted logistic regression model, there was no statistically significant difference between the clinical, anthropometric, and food intake variables according to the diagnosis of sarcopenia between the groups.

**Figure 2:**
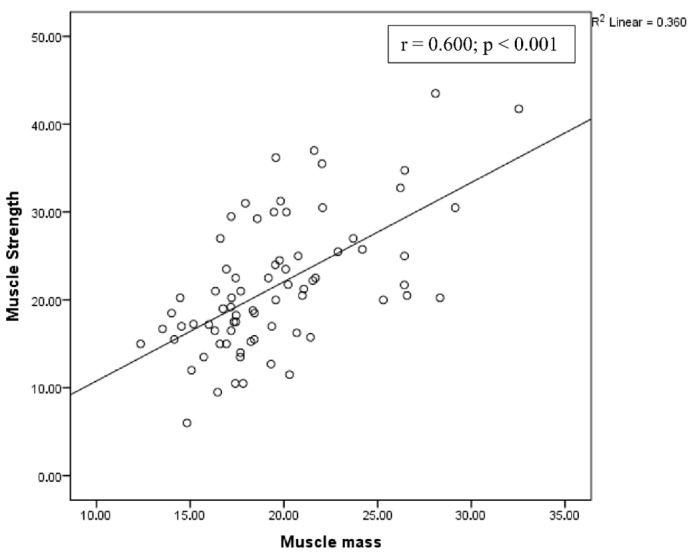
Correlation between muscle strength and muscle mass.

**Figure 3:**
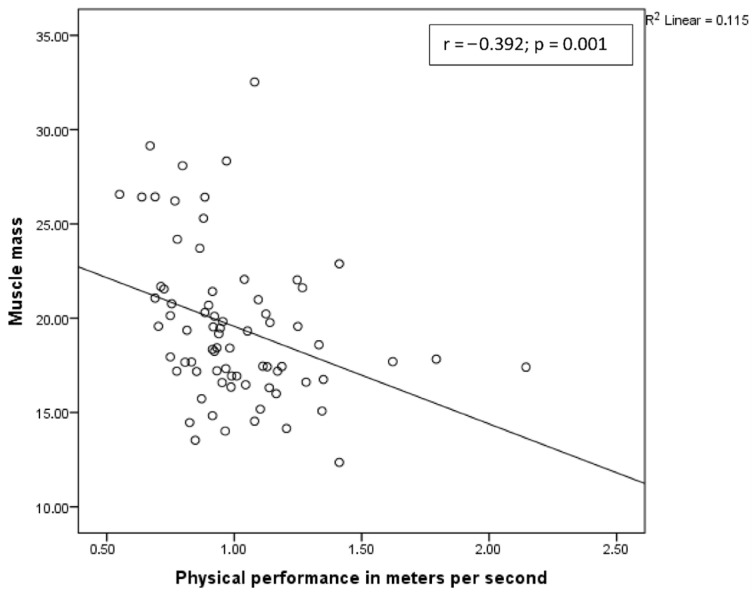
Correlation between muscle mass and physical performance.

A positive correlation was observed between muscle strength and muscle mass, and a negative correlation was observed between physical performance and muscle mass (Figure 2 and Figure 3, respectively).

## 4. Discussion

This sample of patients with MASLD was mostly composed of adult women, those with obesity, and those with metabolic syndrome, and only two patients were diagnosed with sarcopenia. Habitual dietary consumption was similar for most nutrients between individuals without sarcopenia and those with probable sarcopenia. Lower calcium intake was observed in individuals with probable sarcopenia.

Although dietary intake is a known factor that may contribute to the onset of MASLD, the literature findings regarding the dietary patterns of affected individuals show considerable variation in caloric intake, macronutrients, and micronutrients [39,40,41,42]. This inconsistency makes it challenging to identify the specific dietary composition that contributes to the development or progression of the disease.

Dietary consumption did not reveal significant differences across various aspects of MASLD. When assessing the food consumption of individuals with MASLD based on the degree of hepatic steatosis (mild or moderate) [43] and the presence or absence of steatohepatitis confirmed by liver biopsy [44], no variations were observed in total energy, macronutrient, or micronutrient intake between the groups. The dietary habits of individuals with MASLD and sarcopenia remain underexplored in the literature. However, inadequate protein [14] and energy [13] intake have been linked to the isolated presence of sarcopenia components in individuals with MASLD. In the present study, the median protein and calorie intake in both groups fell short of the recommended ≥1.2 g per kilogram of body weight per day [12] and 20–25 kcal per kilogram of body weight per day [26], respectively.

Yi and colleagues [14], who defined sarcopenia based solely on skeletal muscle mass, investigated the relationship between food intake and sarcopenia in patients with MASLD. Their findings suggest that protein intake categorized as adequate (1.2–1.5 g/kg of body weight) may have a protective effect against sarcopenia. However, no associations were identified when carbohydrate, lipid, energy, and protein intakes were assessed in absolute terms. Similarly, Himoto and colleagues [13] did not observe an association between protein intake and skeletal muscle mass in individuals with MASLD. The definition of sarcopenia is crucial for identifying its prevalence and possible causes [45].

In the general population, where more studies are available on this topic, protein and energy intake among individuals with sarcopenia show inconsistent findings. A review by Santiago and colleagues [46] reported that elderly individuals with sarcopenia had lower protein and energy intake compared to their non-sarcopenic counterparts. The meta-analysis results by Almeida and colleagues [47], which utilized the diagnostic criteria for sarcopenia from the EWGSOP in the elderly population, revealed no difference in protein intake between individuals with and without sarcopenia. However, the results showed that patients with sarcopenia consume fewer kilocalories per day than patients without sarcopenia. It is important to consider the possibility that the data reported on food consumption through the 24hR may be underestimated or may be the result of nutritional counseling for MASLD outpatients.

This study appears to be among the first to assess the adequacy of dietary intake in patients with MASLD based on a diagnosis of sarcopenia, following nutritional guidelines. Lower calcium intake emerged as the only nutrient associated with probable sarcopenia. Similarly, reduced calcium intake has been noted in other populations with sarcopenia, such as the elderly [46]. Calcium has a critical role in muscle fibers, as a signaling mineral [48]. Therefore, calcium deficiency can negatively impact muscle mass, strength, and contraction quality, potentially contributing to the development of sarcopenia [49].

Although the results indicated lower calcium intake among patients with probable sarcopenia, it is important to note that calcium consumption in both groups fell below the Recommended Dietary Allowance (RDA) of 1000 mg/day for individuals of both genders [50]. Given the low dietary calcium intake in the study sample, individuals with MASLD should be advised to maintain a diet rich in this nutrient to achieve the recommended daily intake. It is worth noting that, to date, no studies have been found that support the use of calcium supplementation in the MASLD population. In the general population, where there are more studies, there is no evidence of routine calcium supplementation as a means to prevent sarcopenia [51].

The low protein and calcium intake observed in MASLD patients, both those with probable sarcopenia and those without, may be linked to limited income and purchasing power. Protein, calcium, and other essential nutrients are predominantly found in animal-based foods such as meat, fish, milk, and dairy products, which are often financially inaccessible. Beef and dairy consumption are historically lower in the Northeast/Bahia compared to the South/Southeast of Brazil. Furthermore, studies indicate a declining trend in the consumption of beans, a staple food rich in vegetable protein, which is a prominent feature of the dietary culture in Northeast Brazil and Bahia [52,53]. Consequently, the socioeconomic profile of the patients, primarily reliant on public healthcare services, likely contributes to the inadequate intake of these vital nutrients in the evaluated population. Despite the controversial evidence on the role of dairy in the human diet and the lack of data on the effect of milk dietary beliefs on dietary intake in populations, many of our patients report excluding this food group to eliminate the possible inflammatory effect [54].

The presence of sarcopenia and probable sarcopenia in the MASLD population is associated with liver fibrosis [55,56] and its severity [57,58]. Reduced muscle mass and strength increase the risk of liver disease progression, given the chronic inflammation, decreased insulin sensitivity, and mitochondrial dysfunction [6]. Therefore, monitoring of liver fibrosis through non-invasive tools should be used in the follow-up of patients with MASLD and complemented by the assessment of muscle strength, which is the initial measure for diagnosing probable sarcopenia.

In clinical practice, the diagnosis of sarcopenia has become increasingly valued, and sarcopenia has been associated with a poor prognosis. However, in addition to changes in the definition of sarcopenia, which could lead to an overestimation of its prevalence, prevention (probable sarcopenia) may be where we can act most effectively [57,59,60], since we still have no evidence of the role of nutrition in reversing sarcopenia [61].

Sarcopenia is known to have a multifactorial etiology. In individuals with MASLD, potential mechanisms may include insulin resistance resulting from hepatic fat infiltration [62], physical inactivity, and muscle disuse, all of which contribute to decreased muscle function [4]. Lifestyle factors, particularly reduced physical activity, appear to have a stronger association with sarcopenia in MASLD [14]. Exercise plays a crucial role in mitigating this risk by reducing the production of pro-inflammatory cytokines and increasing the production of anti-inflammatory cytokines, thereby enhancing muscle protein synthesis and regeneration, as well as improving glucose uptake [63]. In the studied sample, most individuals were physically active, and only a minority exhibited advanced liver fibrosis. This combination may partially explain the low prevalence of sarcopenia observed in the population.

The present investigation offers valuable insights into the dietary patterns and sarcopenia in individuals with MASLD, utilizing validated methodologies and tools for measuring sarcopenia components. The 24 h dietary recall (R24h) was employed to assess habitual dietary intake, offering advantages such as ease of application, low cost, minimal alteration of food intake, and no requirement for the patient to be literate [64]. Three R24h recalls were conducted at different times to adjust nutrient distribution, remove intrapersonal random error, and estimate habitual intake [65].

Although this research has provided valuable insights, it is important to acknowledge its limitations. The cross-sectional design prevents the establishment of causal relationships, and longitudinal studies are necessary to further investigate the role of dietary intake in the development of sarcopenia in MASLD patients. Additionally, the relatively small sample size limits the ability to generalize the findings to a broader population. The use of the FIB-4 marker, while a non-invasive and practical alternative to liver biopsy, may not offer the same accuracy, as liver biopsy remains the gold standard for assessing fibrosis. Finally, reliance on self-reported dietary intake introduces the risk of recall bias, with the possibility of underreporting, particularly in patients who had previously received nutritional counseling.

Despite these limitations, the study makes an important contribution to the field and highlights the need for further research to understand the complex relationship between diet, sarcopenia, and MASLD.

## 5. Conclusions

In this sample of MASLD patients, only two patients were diagnosed with sarcopenia; lower calcium, energy, and protein intake were observed, but only lower calcium was clinically significant in probable sarcopenia. Thus, it is demonstrated that individuals with MASLD receive guidance on the adequate consumption of foods that are sources of calcium and have their muscle strength assessed during nutritional evaluation, as this parameter enables the early detection of probable sarcopenia cases in this population.

## Figures and Tables

**Table 1:** Classification of the diagnosis of sarcopenia according to the European Working Group on Sarcopenia in Older People, 2019.

Diagnostics	EWGSOP2, 2019
No sarcopenia	MM + MS + PP adequate
Probable sarcopenia	MS insufficient
Sarcopenia	MS + MM insufficient
Severe sarcopenia	MS + MM + PP insufficient

EWGSOP: European Working Group on Sarcopenia in Older Persons; MM: muscle mass; MS: Muscle strength; PP: physical performance.

**Table 2:** Sociodemographic, clinical, and anthropometric characteristics of patients with metabolic dysfunction-associated steatotic liver disease, evaluated according to probable sarcopenia diagnosis.

Variables *n* (%)	Total (*n* = 74)	No Sarcopenia (*n* = 53)	Probable Sarcopenia (*n* = 21)	*p* Value *	Cramér’s V
Female sex	55 (74.3)	40 (75.5)	15 (71.4)	0.72	0.42
Physically active	47 (65.3)	36 (70.6)	11 (52.4)	0.17	0.174
Nutritional monitoring	44 (60.3)	31 (59.6)	13 (61.9)	0.86	0.021
BMI (kg/m^2^)					
Eutrophy	11 (14.9)	8 (15.1)	3 (14.3)	0.99	0.015
Overweight	17 (23.0)	12 (22.6)	5 (23.8)		
Obesity	46 (62.2)	33 (62.3)	13 (61.9)		
High WC (cm) ***	63 (87.5)	46 (86.8)	17 (89.5)	1.00 **	0.036
Metabolic syndrome	48 (64.9)	34 (64.2)	14 (66.7)	0.84	0.024
Degree of steatosis					
Grade I	26 (35.1)	22 (41.5)	4 (19.0)	0.10 **	0.220
Grade II and III	48 (64.4)	31 (58.5)	17 (81.0)		
Degree of fibrosis by FIB-4					
Absence of advanced fibrosis (F0/F1/F2)	51 (69.9)	38 (73.1)	13 (61.9)	0.27	0.191
Presence of advanced fibrosis (F3/F4)	5 (6.8)	2 (3.8)	3 (14.3)		
Indeterminate	17 (23.3)	12 (23.1)	5 (23.8)		

Pearson’s chi-squared test, ** Fisher’s exact test. BMI: body mass index; WC: waist circumference; FIB-4: Fibrosis-4 Index. *** *n* = 72, missing data from two patients. * The sociodemographic, clinical, and anthropometric variables of patients with MASLD were not associated with the absence of sarcopenia or with probable sarcopenia. For the variable sex, the lack of significance may be due to insufficient sample size rather than the absence of an effect (Cramér’s V, 0.3–0.5).

**Table 3:** Characteristics of dietary intake in patients with metabolic dysfunction-associated steatotic liver disease, assessed according to the diagnosis of probable sarcopenia.

Variables **	No Sarcopenia Me [IQR 25; 75]	Probable Sarcopenia Me [IQR 25; 75]	*p* Value ***	r	PS
Energy (kcal)	1587.5 [1441.0; 1902.1]	1511.8 [1344.1; 1878.]	1.00	0.114	0.573
Energy (kcal/kg weight/day)	20.2 [16.1; 23.5]	19.0 [16.7; 23.3]	0.60	0.038	0.525
Carbohydrates (g)	199.4 [173.7; 217.8]	194.1 [178.3; 251.3]	0.60	0.030	0.519
Protein (g/kg body weight/day)	0.8 [0.7; 1.0]	0.7 [0.6; 0.8]	0.52	0.188	0.621
Total fats (g)	64.1 [57.0; 78.9]	58.8 [49.5; 72.5]	0.60	0.174	0.612
Saturated fats (g)	21.1 [18.6; 26.1]	19.1 [15.0; 23.4]	0.12	0.242	0.656
Monounsaturated fats (g)	21.3 [18.0; 27.7]	20.0 [16.4; 25.6]	0.60	0.105	0.568
Polyunsaturated fats (g)	16.0 [13.0; 19.1]	13.8 [11.6; 17.3]	0.30	0.182	0.617
Dietary Fiber (g)	22.0 [18.0; 25.4]	22.1 [18.0; 25.3]	1.00	0.031	0.520
Calcium (mg)	512.6 [444.5; 675.0]	451.8 [389.8; 584.9]	0.04	0.210	0.635
Iron (mg)	8.9 [7.2; 10.6]	9.0 [7.7; 11.0]	1.00	0.056	0.536
Phosphorus (mg)	970.7 [810.4; 1132.7]	929.3 [755.40; 1086.5]	0.60	0.134	0.587
Selenium (µg)	74.9 [61.9; 94.7]	69.5 [59.6; 89.5]	1.00	0.076	0.549
Zinc (mg)	8.6 [7.1; 10.7]	8.5 [7.4; 11.4]	1.00	0.066	0.542
Magnesium (mg)	229.5 [201.6; 287.7]	229.3 [190.9; 266.7]	1.00	0.104	0.567

** Results are presented as Me: median, IQR: interquartile range; *** Mann–Whitney test. r: Rosenthal’s r, PS: probability of superiority.

## Data Availability

The original contributions presented in this study are included in the article. Further inquiries can be directed to the corresponding author.
